# An inverse optimization approach to understand human acquisition of kinematic coordination in bimanual fine manipulation tasks

**DOI:** 10.1007/s00422-019-00814-9

**Published:** 2020-01-06

**Authors:** Kunpeng Yao, Aude Billard

**Affiliations:** Learning Algorithms and Systems Laboratory (LASA), École Polytechnique Fédérale de Lausanne (EPFL), Lausanne, Switzerland

**Keywords:** Human motion understanding, Kinematic coordination, Inverse optimization, Bimanual fine manipulation

## Abstract

Tasks that require the cooperation of both hands and arms are common in human everyday life. Coordination helps to synchronize in space and temporally motion of the upper limbs. In fine bimanual tasks, coordination enables also to achieve higher degrees of precision that could be obtained from a single hand. We studied the acquisition of bimanual fine manipulation skills in watchmaking tasks, which require assembly of pieces at millimeter scale. It demands years of training. We contrasted motion kinematics performed by novice apprentices to those of professionals. Fifteen subjects, ten novices and five experts, participated in the study. We recorded force applied on the watch face and kinematics of fingers and arms. Results indicate that expert subjects wisely place their fingers on the tools to achieve higher dexterity. Compared to novices, experts also tend to align task-demanded force application with the optimal force transmission direction of the dominant arm. To understand the cognitive processes underpinning the different coordination patterns across experts and novice subjects, we followed the optimal control theoretical framework and hypothesize that the difference in task performances is caused by changes in the central nervous system’s optimal criteria. We formulated kinematic metrics to evaluate the coordination patterns and exploit inverse optimization approach to infer the optimal criteria. We interpret the human acquisition of novel coordination patterns as an alteration in the composition structure of the central nervous system’s optimal criteria accompanied by the learning process.

## Introduction

Bimanual coordination is central to humans’ daily activities and to most craftsmanship. Tasks such as lacing shoes and knitting fabric can hardly be accomplished using one single hand. Human hands and arms are endowed with more than thirty degrees of freedom (DoFs). This is far more than is required when controlling end-point motion in a 6-DoF space. Our upper limbs are hence highly redundant motor systems. Yet, humans display very consistent kinematic patterns when performing the same task, seemingly making little use of this redundancy (Morasso 1983). A wealth of evidence speaks in favor of the hypothesis that the central nervous system (CNS) overcomes the inherent motor redundancy and masters excessive degrees of freedom through a synergistic coordination of muscles and joints (Bernstein 1967), resulting in stereotyped movements (Flash 1990). Each task, however, may require a different synergistic coordination. The process by which these coordination patterns are acquired remains poorly known, especially in bimanual tasks.

### Coordination patterns in upper limb motions

Coordination patterns in upper limb motions have been intensively investigated. Studies on arm-hand coordination are primarily within the context of reach and grasp movement (Hoff and Arbib 1993). These studies revealed phase relationships between hand aperture/closure and arm movements, indicative of temporal synergies between arm and hand. When the reach and grasp movement is perturbed, e.g., when the target is moved during the movement, it was shown that these phases were modulated by the change in target position, and are hence anchored on the target motion (Jeannerod et al. 1995). Vainio and Tiainen (2018) discovered a systematic interaction in proximal and distal prehensile components between two hands, which may be related to the motor preparation of arm extension and grasping motion.

A large amount of studies focused on the inter-limb coordination patterns in symmetric (Kelso 1994) and rhythmic arm motions, such as drawing circles in symmetric and asymmetric manners (Semjen et al. 1995; Cattaert et al. 1999; Franz and Ramachandran 1998). Experimental results suggest that the bilateral motions of limbs during everyday activities manifest trend toward in-phase (homologous muscle groups activate simultaneously) and anti-phase (homologous muscle groups activate in alternation) coordination patterns (Howard et al. 2009), and upper limb motions are more stable if their frequency ratio is an integer instead of non-integer (Summers et al. 1993). It is also suggested that same amplitudes and isotropic directions may be the default mode of bilateral motions, and assimilation effects are likely to occur otherwise (Swinnen and Gooijers 2015).

A few works have focused on the coordination patterns underlying asymmetric and non-cyclic motions, such as asymmetric reaching motion toward distinct targets (Kelso et al. 1979; Bingham et al. 2008). It is in a way surprising that so little attention has been brought to this topic, since such bimanual movements are quite common in human everyday activities (e.g., picking-up large objects). These studies confirmed the existence of bimanual coupling. For instance, reaching tasks are performed more easily when both hands reach the targets simultaneously. When the two hands are involved in asymmetric reaches, the movements of the hand reaching for the target farthest away influences the movement of the other hand, slowing it down to preserve temporal coordination (Bingham et al. 2008).

### Human selection of hand poses for tool use

In comparison with arms, human hands have even more degrees of freedoms that result in much higher redundancy. It has been suggested that the CNS reduces the complexity of controlling fingers by selecting proper hand poses to grasp or to manipulate objects in a purposeful way, through task-oriented grasps Rizzolatti et al. (1988). Several taxonomies (Kamakura et al. 1980; Cutkosky et al. 1989; Feix et al. 2015) have been offered to categorize the large numbers of grasps used in everyday life, and multiple measures for determining the appropriateness of the grasp have been proposed (Ferrari and Canny 1992; León et al. 2012). These measures enable evaluation of how well the grasp may stabilize the object or enable the subsequent manipulation (Endo et al. 2007). A proper hand pose guarantees transmission of force or generation of torque demanded by the task, and the placement of fingers enables the ability of holding tools and performing manipulation.

A wealth of evidence shows that humans have already selected the appropriate hand pose prior to closing the fingers on the object. While the arm moves toward the object, the hand pre-shapes the fingers toward the final finger configuration. The formation of hand poses demands coordination cross many DoFs of fingers, and depends highly on the grasping purpose (Dollar 2014) and target objects’ properties (Bullock et al. 2013; Feix et al. 2014).

A popular approach to explain the coordination mechanism underlying finger control is the concept of synergies (Santello et al. 1998), which describes the coupling of multiple finger joints in a low-dimensional space. Hand poses may hence be generated from a single or a combination of multiple synergies. The analysis of hand posture synergy is commonly conducted using principal component analysis for static hand poses (Santello et al. 1998) or singular value decomposition for temporal sequences (Mason et al. 2001). However, the selection of hand pose is not only based on the functionality of the grasp objective (e.g., generating force or torque for task execution), but also highly related to the subjects’ perception of task demands (Friedman and Flash 2007).

Traditional synergy-based analysis that exploits only kinematic information does not explain how grasps respond to task demands (Ekvall and Kragic 2005), as the hand pose does not contain information on the tool, nor on the force applied. This refers to grasp on tools that need to exert specific pattern of forces during subsequent manipulation. One approach to relate grasps to manipulation request was done by Friedman and Flash (2007). Task compatibility of selected human hand poses are evaluated based on velocity, force transmission ellipsoids and stiffness ellipsoids. In our previous work, we also offered a mean to categorize grasps (De Souza et al. 2015) by combining tactile and postural information of human grasp, and related this to the opposition space theory (Iberall 1987). These approaches, however, do not explain how humans can modulate their grasps to better account for task demands. In the present study, we study how hand poses reflect the coordination patterns and control strategies that humans exploit for tool manipulation. We hypothesize that hand poses vary not only for manipulating distinct tools, but also as a result of proficiency. We analyze subjects’ finger placements on the tools and compare hand poses, aiming to infer the differences in the understanding of task demands for subjects of different skill levels.

**Fig. 1:**
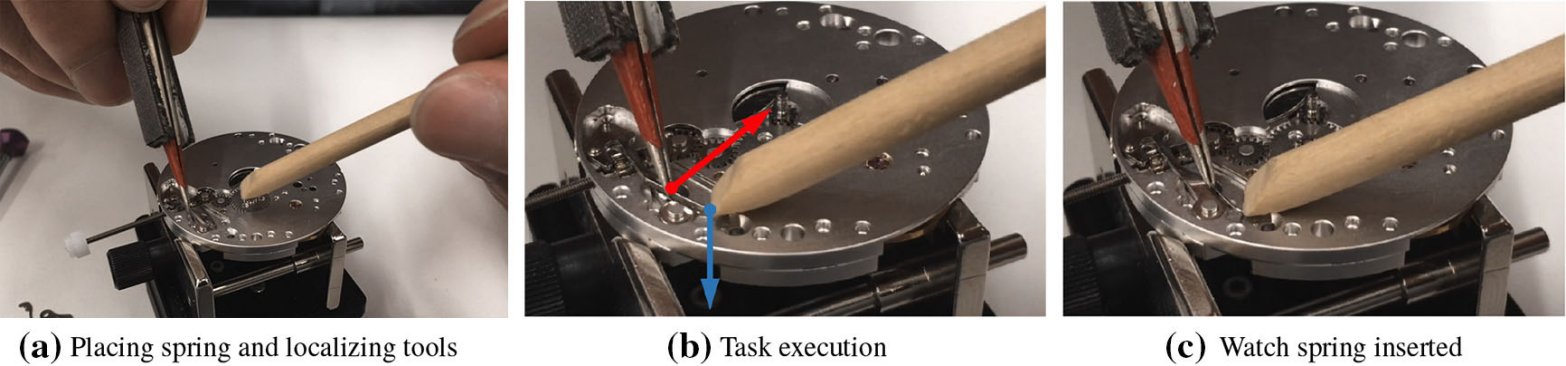
Steps of inserting the watch spring (pictures were taken in front of the subject). The left hand holds a pegwood, and right hand manipulates a pair of tweezers. **a** The spring is unmounted. Subject picks up the watch spring using tweezers and places it onto the watch face. **b** Subject localizes the tools onto the watch face: the pegwood (left) presses the watch spring downwards to maintain it stable; in the meanwhile, the tweezers pinch the foot of the spring. **c** Subject pushes the tweezers to compress the foot of watch spring for insertion

This study investigates coordination of arms and human selection of hand poses in an asymmetric and non-cyclic bimanual task. We focus on a typical task in watchmaking manipulation—the insertion of a watch spring (see Fig. 1). To perform the task, the two hands manipulate two different tools, tweezers and a wooden stick (pegwood). They are constrained spatially to work on the same piece and follow also temporal constraints to move in synchronization to insert the spring. Studying the posture of the arms and fingers on the tools as well as the relative movements of the tools allow to determine the asymmetrical and non-cyclic nature of the coordination patterns in such bilateral motions.

### Acquisition of coordination patterns

The question of whether the coordination patterns reviewed above are “innate” or learned remains open. One of the earliest coordinated movements, kicking action (Thelen 1985), can be traced back to the intrauterine stage. Fetuses in the womb display goal-directed kinematic patterns in hand motions by 22 weeks of gestation (Zoia et al. 2007). Coordination patterns between separate motor systems such as hand-to-mouth coordination (Butterworth and Hopkins 1988) and hand-to-eye coordination (Von Hofsten 1982) are also observed at birth. Studies of infants reaching motion, however, suggest that the complex visuo-motor coordination patterns required to control precisely for arm and hand to grasp objects are acquired through a learning process (Georgopoulos et al. 1981; Konczak et al. 1995). Non-random changes in hand and digit movements are observed in human fetus (Sparling et al. 1999). Shortly after birth, infants manifest early grasping behaviors (Twitchell 1970). Around 4–5 months, infants start to demonstrate typically successful grasping motion (Sgandurra et al. 2012), and the coordination between arm extension and finger flexion is also observed (Schneiberg et al. 2002). Yet, little is known on how humans learn coordinated control for both force and kinematic of hand movements to perform fine manipulation tasks, such as playing musical instruments (Furuya et al. 2011; Jerde et al. 2012).

Approaches to explain human acquisition of coordination patterns take either an information-processing point of view or a dynamic-pattern perspective (Swinnen and Gooijers 2015). The information-processing theoretical framework suggests the main barrier in acquiring bimanual coordination patterns lies in the interference between limbs, i.e., the so-called neural cross talk (Marteniuk et al. 1984; Heuer et al. 2001). Through practice or the integration of sub-tasks, humans are able to overcome the constraints produced by the above-mentioned factors, so as to decouple the task in asymmetric motions with distinct amplitude and directions of both arms and learn a new bimanual skill (Swinnen et al. 1993). However, it has been argued that bimanual coordination is instead the “default mode” of the control system (Swinnen and Gooijers 2015) with uni-manual action being rather only a suppression of the bimanual coordination nature of human motion. The dynamic-pattern framework (Schöner and Kelso 1988; Kelso 1997) considers the human acquisition of skills as an alteration of the intrinsic patterns (e.g., physical constraints, prior knowledge, and experience) under behavioral information (e.g., constraints imposed by task and environmental) (Schöner et al. 1992). It is suggested that human motor system generates bimanual coordination patterns as a result of the interaction of constraints at various levels (Swinnen 2002; Swinnen and Gooijers 2015). Studies also confirmed that the acquisition of novel coordination patterns is affected by a variety of factors, such as phase (Haken et al. 1985), direction (Baldissera et al. 1982; Swinnen et al. 1997, 1998), timing (Yamanishi et al. 1979; Deutsch 1983; Peters 1981), and amplitude (Franz 1997; Spijkers and Heuer 1995). It shows that the accuracy requirement of task reduces relative phase bias in the bimanual coordination patterns (Wang et al. 2017). Visual guidance plays also an important role, as it reduces the complexity of bimanual coordination (Vaz et al. 2017). Coordination patterns can be acquired through training, and practice could enhance the stability of the acquired coordination patterns (Smethurst and Carson 2001). Notwithstanding the foregoing, it remains unclear what task-specified coordinated patterns do humans generate and also whether the coordination patterns change as human subjects continue practice and improve task proficiency, i.e., whether these factors have consistent impact on the generation of coordination patterns at different learning stages.

### Optimal control theory for human motion understanding

A central problem in motor control is to understand how humans coordinate redundant degrees of freedoms to generate movements. Optimal control theory (Engelbrecht 2001; Todorov 2004) considers the human motion generation as an optimization process and suggests that the CNS may optimize underlying optimal criteria (or cost functions) in order to reduce the control complexity by means of coordination. It is presumed that the underlying optimal criterion of human CNS is composed of multiple weighted costs, such as energy expenditure of both inertial and gravitational forces (Berret et al. 2008). Therefore, inferring the optimal criteria used by the CNS is key to understanding the mechanism of human motion. This becomes an inverse problem to human motion planning and generation.

Inverse optimal control (IOC) can be used to infer the weights of corresponding costs that compose the optimal criteria of CNS. For instance, Mombaur et al. (2010) used IOC to determine the optimal criteria for human to generate locomotion. To analyze arm motion, Berret et al. (2011) provides experimental evidence for the existence of composite cost for CNS and suggests that human CNS is trying to minimize mechanical energy expenditure ($40\%$ on average) and joint level smoothness ($35\%$ on average) during arm reaching motion. Mainprice et al. (2015) applied IOC to analyze human arm reaching motion in an interaction task and suggests that humans try to increase the smoothness of arm trajectories rather than to avoid collision with other human subject. Oguz et al. (2018) used IOC to investigate the trade-off between kinematics-based and dynamics-based controllers depending on the reaching task. These works focus on modeling human motions as displayed by adults and not on modeling the process by which these motions have been acquired. They have not taken subjects’ proficiency into consideration. Moreover, constraints imposed by task demands are not considered.

The present study follows an optimal control framework to interpret human kinematic coordination paying particular attention to proficiency of subject. Unlike the above-mentioned studies that exploit IOC to analyze the process of generating optimal motion trajectories, we focus on understanding the planning of the generated kinematic postures and thus formulate it as an inverse optimization problem (IOP) without considering the control input (e.g., joint torque) of the human arm. Furthermore, We take into account modeling and acquisition of task demand explicitly within the optimization process of the CNS. Indeed, as subjects acquire a skill, they acquire simultaneously a model of the task demands and of the pattern of hand-arm coordination that can best meet these task demands. Aside from the inherent constraints of human body, such as muscle/bone capability and musculoskeletal structure, the ability to generate forces to meet task demands is also highly affected by the kinematic properties (Chiu 1987). We, hence, consider among potential task-level costs that CNS may try to optimize, the singularity of posture, ability to satisfy task demands, and also comfort—from a biomechanical point of view. Our optimization framework is hence a composite of optimal criteria, which includes both inherent (e.g., biomechanical constraints) and extrinsic (e.g., constraints imposed by task demands) costs.

The composition structure of CNS’s optimal criteria is indicated by the weights attached to the different cost components. Taking advantage of inverse optimization approach, we infer the weights of these cost components to investigate the composition structure of this optimal criterion. We hypothesize that the different coordination patterns across novice and experts may be revealed by different weights associated with each cost. These weights change as skill improves. We, hence, compare the composition structures of CNS’s optimal criteria of human subjects at different skill levels, so as to decipher how such bimanual coordination patterns are generated with improvement in task proficiency.

**Fig. 2:**
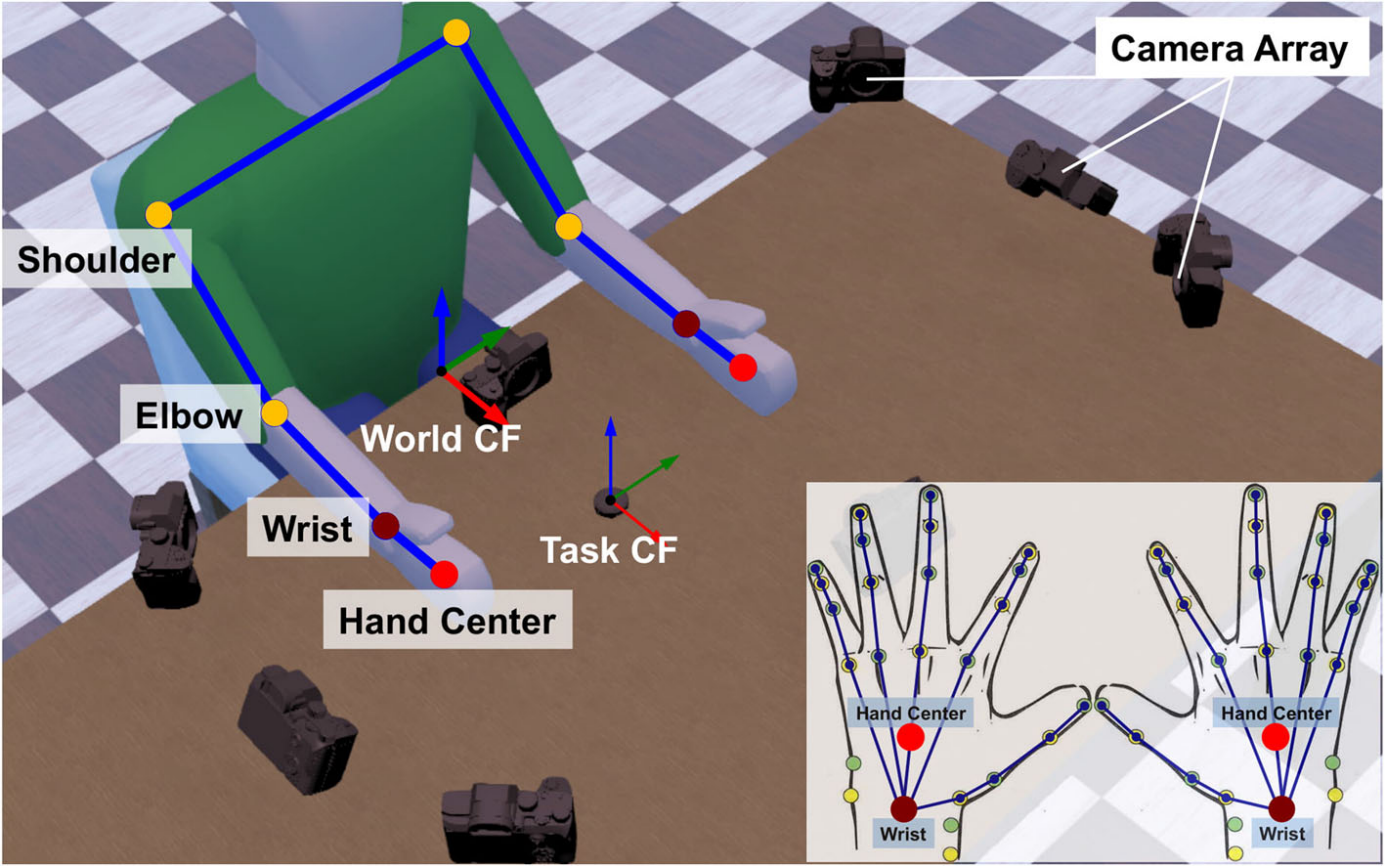
Experimental scenario and human skeleton model. The end-effector coordinate frame of the arm model (upper left) overlaps the hand coordinate frame (bottom right)

## Methods

To study bimanual coordination, we requested subjects to perform a typical watchmaking task that requires precise inserting and loading the watch’s spring (a key element to the functioning of the watch). Steps of performing the spring assembly task are explained in details in Sect. 2.3.1. Statistics are presented in the form of mean ± std values.

We collected both tactile information (applied force) and kinematic information on hands and tools (Sect. 2.2). From recorded force information, we extracted task demands (Sect. 2.4); and from the recorded videos, we reconstructed skeleton models of arm and hand (Sect. 2.5). We computed hand pose on manipulating tools and express these as coordinated patterns of finger joints. We formulated metrics (Sect. 2.6) to evaluate kinematic coordination patterns of human hands and arms. We developed a model based on inverse optimization to explain differences across novice and experts (Sect. 2.8).

### Subjects

Fifteen subjects took part in the study. They were recruited from ETVJ (École Technique de la Vallée de Joux) on a voluntary basis following wide announcement. The ability to mount the spring of the watch is acquired during the first 2 years of the study at ETVJ and is hence considered as mastered for students in the 3rd year and above. Hence, subjects were assigned to novice group (ten) and expert group (five) according to the number of years of practice at watchmaking. Novice subjects (nine men, one woman; age range 16–30, mean ± std: $18.60\pm 4.33$) comprised students in first year of study at ETVJ with no prior expertise at watchmaking. Experts (three men, two women; age range 17–36, mean ± std: $21.80\pm 7.98$) encompassed four students in 3rd and 4th year of study and one professional, all of whom had more than 24 months of practice at watchmaking. All subjects were right-handed.

The experimental protocol was approved by the Human Research Ethics Committee of EPFL. All subjects were provided with an information sheet, and they (or their caretaker when underage) gave informed consent prior to the experiments. Subjects received detailed verbal instructions prior to starting the experiment. They were given the opportunity to ask as many questions as they wished and to train themselves at performing the tasks while wearing the tactile glove (which partially hindered natural sense of touch). No further instructions regarding task objective or performance evaluation were given to the subjects, and they were left free to perform the task.

### Apparatus

Workspace is defined as a rectangular-shaped area (50 cm $\times$ 50 cm) on the table surface and in front of the seated subject (see Fig. 2). The manipulation platform—watch face, is fixed on a cuboid plastic base with a miniature force/torque sensor (ATI Nano17) mounted underneath. The base is placed in the center of the workspace facing the subjects at the start of each experiment. Subjects are free to change the pose of the base.

Eight GoPro cameras are arranged in a rectangular formation around the subject’s workspace. Redundancy across angle of view ensures that one can capture the motion of the subject’s arms and hands, the movement of the tools (pegwood and tweezers) and watch base from multiple angles. Twenty-five colored dots (trackers) are pasted on each hand of human subject to track their hand poses and fingers motions: four trackers on each finger, one on the hand center, four around the wrist (two near the tail of ulna and two near the tail of radius, see the bottom right figure of Fig. 2). Wrist joint position is estimated as the center of the ulna-radius trackers. The pose of watch face is tracked by localizing its center and the crown of watch stem manually in the image and then use a smooth tracker across the flow of image. Tools used by both hands (pegwood and tweezers) are tracked through the same procedure by localizing their tails. Movements of tracked positions are recorded by the camera array, and their trajectories are extracted from the recorded videos afterward. Shoulder (glenohumeral joint) and elbow positions are tracked during the post-processing of recorded videos, by manually adding trackers near the real joints on the video frames, based on visual observation.

**Fig. 3:**
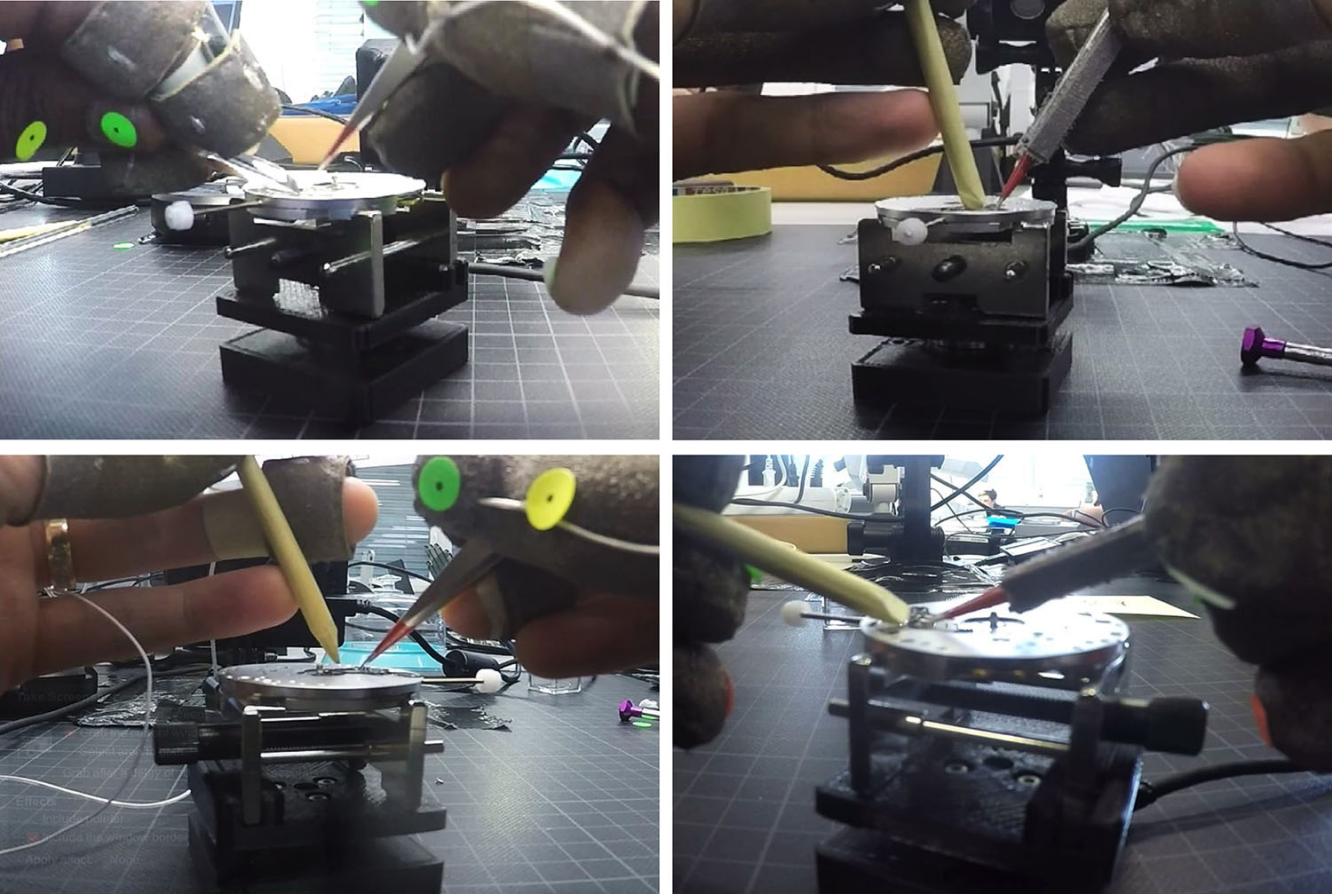
Illustration of subjects rotating watch face during watch spring assembly

We define two Cartesian coordinate frames: the world coordinate frame ${\mathbb {W}}_{\mathrm {CF}}$, located in the middle of the workspace’s border; and the task coordinate frame ${\mathbb {T}}_{\mathrm {CF}}$, located in the center of watch face^1^ (see Fig. 2). The ATI sensor records the force and torque applied to watch face at 70Hz, and GoPro cameras record videos at 50Hz.

### Study design and data collection

#### Task description

Subject was asked to sit in front of the table on a stool. The table and stool are part of the regular furniture used by students and teachers at ETVJ for practice session. All sensors were mounted and calibrated before the experiment starts. Subject was allowed to freely move the watch face and tools (see Fig. 3). At the beginning of each experiment, the subject was asked to assemble and disassemble several components on the watch face, in order to get familiar with the experimental apparatus. This manipulation trial was discarded in the analysis reported. After this initial familiarization phase, the subject was asked to assemble the watch spring. No time pressure was put on the subject, and each subject performed the task at his own pace.

Insertion of the watch spring is performed as follows. The subject first places the watch spring on its target position on the watch face (Fig. 1a). The subject’s assistant hand (left hand in our study) holds a pegwood and presses the watch spring in order to maintain it in place as the other hand loads it. To load the spring, the subject’s dominant hand (right hand in our study) uses the tweezers (with the end closed) to push the foot of the spring so that it falls into the reserved slot (Fig. 1b). Failure happens if the spring was not inserted or not correctly loaded. The manipulation is also considered failed if the watch spring drops or if it flies away during loading. Subjects were allowed to try again in case of failures. A trial ends only once the watch spring is stably inserted (Fig. 1c). Each subject was asked to repeat the spring insertion six times. The first two trials were discarded as the subject was still familiarizing him/herself at doing the task with the sensorized gloves.

#### Motion segmentation and data selection

The recorded manipulation trial was further cut into multiple motion segments. Each motion segment contains one swipe attempt at inserting the spring. It starts when both tools are located at their respective target positions on the watch face and the subject is about to press the tweezers to close them prior to the assembly execution (see Fig. 1b). The segment ends when the spring is released from the tweezers after pressing on it, regardless of whether the attempt was successful or failed (Fig. 1c). Posture adjustments such as changing arm pose, readjusting finger placements, and rotating watch face were excluded from the data analysed here. Segments that include apparatus malfunctions, irrelevant motions (e.g., unexpected actions), and non-standard operations (e.g., subject uses single hand or odd hand gestures to complete the bimanual task) were discarded. Around $18\%$ of the raw motion segments were discarded from the novice group and $20\%$ from the expert group. After selection, each novice subject has 12.30 segments and each expert subject has 11.20 segments on average.

The average time length of segmented motion piece is $4.33s\pm 2.17s$ for novice group, and $2.98s\pm 1.72s$ for expert group. Success rate of each subject is calculated as the number of success motion segments divided by the total number of motion segments. Analysis of the motion segments indicates a higher [$F(1,13)=15.86,~p<0.01$] success rate of subjects from expert group ($83.49\%\pm 11.97\%$) than the novice group ($57.53\%\pm 11.87\%$) on average.

#### Extraction of kinematic information

To analyze the coordination patterns across the two arms and hands, we extracted the postures of all limbs from the videos recorded by the GoPro cameras. The camera system is calibrated by computing the intrinsic (Zhang 2000) and extrinsic camera matrices (Lepetit et al. 2009) using checkerboard images across the field of view. The location of marker in the workspace is computed by triangulating their coordinates in cameras for which the view of the marker is not obstructed. To extract the 3D marker position at one frame of recording, human operator marks the marker position on the images captured by at least two cameras. The marked coordinates are first un-distorted using the intrinsic camera matrices and then projected to a 3D line by the extrinsic parameter matrix. The 3D position of the marker is constructed by computing the closest point to the 3D lines from all cameras that has a view of this marker. This process is repeated for all markers on the two hands of each participant, in order to record the fingers’ posture. All implementations are developed using OpenCV. Errors of this system comprise both (1) reconstruction error ($3.5\pm 1.4$ pixels on average, tested on collected known points), stemming mainly from the calibration; and (2) marking error (0.85 mm ± 0.15 mm on average, tested on randomly selected markers), i.e., error between manually marked position and its reconstructed value. We obtain the average error as 1.63 mm ± 0.4 mm per marker, by combining both error sources.

Posture of the arm joint was done by hand labeling with an expert tracing the posture in the camera image. Since the arm barely move during the experiment, this is sufficient to obtain accurate estimation. Reconstruction error of the arm joints mainly comes from imprecise visual estimation of the human operator. Approximating the upper limbs as cylinders, the maximum error range of arm joints estimation is 2.44 cm, calculated using the mid-upper arm circumference value (Preedy 2012).

Since the assembly motion requires only a millimeter-scale movement of the tweezers’ tip, we consider the postures of the upper limbs as quasi-static during motion execution. Therefore, we extract subjects’ postures as the subject is about to start the insertion motion (Fig. 1b), which can be considered as the average position over the course of the motion by approximation. Extraction of the posture is done by manually marking and estimating the 3D coordinates of these points of interest from recorded video frames, as described above. After extraction, we have one complete set of tracker positions (62 trackers in total) that describe the kinematic information of each subject, including 3 points on each arm, 25 points on each hand, 2 points on the watch face, 2 on the pegwood, and 2 on the tweezers. Using these extracted points, we were able to reconstruct the posture of both upper limbs, as well as the position/orientation of the watch face and tools.

### Extraction of task demands

It is difficult to construct an analytic model of the task, due to the nonlinear shape of the spring. Instead, we use the motion data (both sensor signals and videos) recorded from expert subjects as ground truth, to (1) extract task demands, and to (2) evaluate the task performance of novice subjects.

We fit the 3D force signal sequence recorded by ATI F/T sensor during each motion segment as one force ellipsoid. The major axis of this force ellipsoid indicates the task-demanded direction of force application. We constructed force ellipsoids of all successful motion segments recorded from expert subjects. The average direction of their major axes is used as the optimal task-demanded force direction $\varvec{f}^{*}$. The task requires left hand to hold the pegwood and exert pressure vertically (blue vector in Fig. 1b) for maintaining the stability of spring, while right limb to manipulate tweezers for pinching the spring and pushing it horizontally (red vector in Fig. 1b). Hence, we decomposed $\varvec{f}^{*}$ in ${\mathbb {T}}_{\mathrm {CF}}$ according to the functionality of each tool: the projection along $\mathrm {Z}_\mathrm {T}$ is used as the task-demanded vector for pegwood ($\varvec{f}^{*}_\mathrm {Z}$, left side); and the projection on the $\mathrm {X}_\mathrm {T}-\mathrm {Y}_\mathrm {T}$ plane is considered the task-demanded vector for tweezers ($\varvec{f}^{*}_\mathrm {XY}$, right hand).

### Modeling of human upper limb

The skeleton model of human upper limbs (see Fig. 2) is defined in the world coordinate frame ${\mathbb {W}}_\mathrm {CF}$ and overlaps with the upper limb of recorded subject. It consists of hand models and arm models of both sides.

#### Skeleton model of human arm

Each arm model (left and right) comprises three links (upper arm, forearm, and hand) and three joints (shoulder, elbow, and wrist) (Wu et al. 2005). Only joint rotations are modeled. Upper arm is linked to the human torso at the shoulder joint, which has three DoFs: arm ab/adduction, circumduction, and extension/flexion. Upper arm and forearm are connected by the elbow joint (one DoF: forearm extension/flexion). The wrist joint connects forearm and hand and has three DoFs: extension/flexion, pronation/supination, and ulnar/radial deviation (Bajaj et al. 2015). Hand link of the arm model is modeled as a constant link attached to the wrist joint. Fingers are not modeled. An end-effector’s coordinate frame is attached at the end of the hand link. It coincides with the base coordinate frame of the hand skeleton model (see Sect. 2.5.2).

The length of each model link was calculated as the distance between the two tracker positions attached at both ends of the link. For example, the length of upper arm is the distance between shoulder joint and elbow joint; the length of forearm is the distance between elbow joint and wrist joint. In particular, the length of the hand link is assigned as the distance from wrist joint to the center of the hand (the tracker attached on the center of the palm, see Fig. 2). Since the joints of human arm and the joints of the constructed arm model do not have a bijective mapping, a point-to-point mapping approach (Peer et al. 2008) is used to fit the arm model to the recorded human arm posture. Extracted positions of joint trackers (shoulder, elbow, wrist, and palm center) are used to solve an inverse kinematics (IK) problem to obtain joint angles for each DoF of the arm model. Joint value of each DoF is bounded within the corresponding anthropomorphic motion range (Preedy 2012). Unique IK solution is guaranteed by setting constraints on both joint position ranges and link postures.

#### Skeleton model of human hand

We used a modified 20-DoF paradigmatic hand model (Malvezzi et al. 2015) to analyze the kinematic coordination patterns of human hands (see Fig. 2, bottom right). The thumb finger has three joints: the distal interphalangeal (DIP) joint (1 DoF: flexion/extension), the MCP joint (1 DoF: flexion and extension), and the carpometacarpal (CMC) joint (2 DoFs: flexion and extension, ab/adduction). Each one of the other four fingers has four DoFs: the ab/adduction and extension/flexion of the MCP joint, the extension/flexion DoF of both proximal interphalangeal (PIP) joint and DIP joint. The length of each finger phalanx is calculated as the distance between the two joints located at both of its ends. Each joint angle of the model is calculated directly using the extracted tracker position. A joint-to-joint mapping approach (Bouzit 1996) is used to fit the hand kinematic model to the recorded hand data. Contacts are defined as points on the surface of finger phalanx and manually estimated from the recorded videos. Soft contact model is used.

### Metrics

To measure the goodness of the control, we considered three factors: (1) a measure $\omega$ of how far the joints are from singularities, (2) a task compatibility measure $\alpha$, that assesses the capability of the upper limb to satisfy task demands, namely to generate the desired force/motion, and (3) a comfort measure $\epsilon$ that estimates potential discomfort that could arise from tension in tendons and muscles caused by certain joints movement.

*Singularity measure*$\omega$ We used the manipulability index proposed by Yoshikawa (1984) as a metric of the overall kinematic configuration quality. Manipulability index corresponds to the volume of manipulability ellipsoid. The Jacobian $\varvec{J}$ maps the joint space velocity $\dot{\varvec{\theta }}$ to the Cartesian space velocity $\dot{\varvec{x}}$ as $\dot{\varvec{x}} = \varvec{J}\dot{\varvec{\theta }}$. A unit sphere in joint space, described by $\big |\big |\dot{\varvec{\theta }}\big |\big |^2 = 1$, can be mapped to an ellipsoid in Cartesian space as:

1 $$\begin{aligned} \big |\big |\dot{\varvec{\theta }}\big |\big |^2 = \dot{\varvec{\theta }}^{\intercal }\dot{\varvec{\theta }} = \dot{\varvec{x}}^{\intercal } (\varvec{J}^{\dagger })^{\intercal } \varvec{J}^{\dagger } \dot{\varvec{x}}^{\intercal } = \dot{\varvec{x}}^{\intercal } (\varvec{J}\varvec{J}^{\intercal })^{\dagger } \dot{\varvec{x}} = 1. \end{aligned}$$

The ellipsoid defined by $\varvec{J}\varvec{J}^{\intercal }$ reveals the flexibility of motion in the task space, and its volume is used as the manipulability measure:

2 $$\begin{aligned} \omega =\sqrt{\det (\varvec{J}\varvec{J}^{\intercal })}. \end{aligned}$$

Maximizing $\omega$ prompts the joints to move toward configurations that are far away from singularities, thus guarantees higher motion flexibility.

*Task compatibility measure*$\alpha$ Assembly task requires applying force in desired direction. Hence, we used force transmission ratio (Chiu 1988) as a task compatibility measure. The relation between Cartesian force space and joint torque space via the Jacobian $\varvec{J}$, $\varvec{\tau } = \varvec{J}^{\intercal } \varvec{f}$, maps a unit sphere in joint torque space to Cartesian force space as a force ellipsoid:

3 $$\begin{aligned} ||\varvec{\tau }||^2 = \varvec{\tau }^{\intercal }\varvec{\tau } = \varvec{f}^{\intercal }(\varvec{J}\varvec{J}^{\intercal })\varvec{f} = 1. \end{aligned}$$

Let unit vector $\varvec{u}$ denote the direction of interest, then the force transmission ratio $\alpha$ is defined as:

4 $$\begin{aligned} \alpha = (\varvec{u}^{\intercal }(\varvec{J}\varvec{J}^{\intercal })\varvec{u})^{-1/2}. \end{aligned}$$

A physical interpretation of $\alpha$ is to consider the distance from the center of the force ellipsoid to its surface, along the direction of $\varvec{u}$. A large value of $\alpha$ implies that for the same joint torque, the arm (or the hand) is capable of applying large force to the environment. In our study, we used the extracted task demands ($\varvec{f}^{*}_\mathrm {XY}$ and $\varvec{f}^{*}_\mathrm {Z}$) as the vector $\varvec{u}$ for both sides, respectively.

*Comfort measure*$\epsilon$ Large joint movements may stretch tendons and lead to discomfort feelings. The closer the movement of the joint-to-joint limit, the less comfort. We adapted the comfort index (León et al. 2012, 2014) to measure the deviation of joint angles from joint position when the hand is in its rest posture, as an indication of joint stretch and associated joint comfort feeling:

5 $$\begin{aligned} \epsilon = 1-\frac{1}{N_J}\sum _{i=1}^{N_J} \bigg (\frac{q_i - q_i^r}{Q_i}\bigg )^{2},~i=1,2,\dots ,N_J. \end{aligned}$$

The angle of $i\mathrm{th}$ joint is $q_i$, and $q_i^r$ denotes the angle when the $i\mathrm{th}$ joint is at rest position. $N_{J}$ denotes the number of joints taken into calculation. This deviation is normalized by the joint motion range $Q_i$, which measures the distance between $q_i^r$ and the joint limit.

### Adaptation of kinematic measures to human model

#### Adaptation to human hand model

We adapted the above-defined measures to the human hand model, to evaluate the hand poses of recorded subjects. Hand singularity measure $\omega ^h$ is modified as:^2^

6 $$\begin{aligned} \omega ^{h}=\sqrt{\det (\varvec{H}\varvec{H}^{\intercal })}. \end{aligned}$$

The hand-object Jacobian $\varvec{H}$ (Shimoga 1996) transmits the applied joint torque $\varvec{\tau }$ to the applied force $\varvec{f}$ in an object reference frame as $\varvec{\tau }=\varvec{H}^{\intercal }\varvec{f}$. It indicates the volume of manipulability ellipsoid (Murray 2017) and reveals the ability of the hand to manipulate the tool. The task compatibility measure is reformulated as:

7 $$\begin{aligned} \alpha ^{h} = (\varvec{u}^{\intercal }(\varvec{H} \varvec{H}^{\intercal })\varvec{u})^{-1/2}. \end{aligned}$$

The direction of force that corresponds to task demand is projected into hand’s coordinate frame and used as vector of interest $\varvec{u}$. The comfort measure of hand is then calculated as follows:

8 $$\begin{aligned} \begin{aligned}&\epsilon ^{h} = 1-\frac{1}{N_f N_{J}^{f}}\sum _{f=1}^{N_f}\sum _{j=1}^{N_{J}^{f}}\bigg (\frac{q_{f,j}-{q}_{f,j}^{r}}{Q_j^f}\bigg )^{2},\\&j=1,2,\dots ,N_{J}^{f},~f=1,2,\dots ,N^{f}. \end{aligned} \end{aligned}$$

The angle of $j\mathrm{th}$ joint of the $f\mathrm{th}$ finger is $q_{f,j}$, and the distance between its rest position ${q}_{f,j}^{r}$ and its joint limit is $Q_j^f$. $N^f_{J}$ denotes total number of joints of $f\mathrm{th}$ finger ($N^f_{J}=4$ in this study), and $N_f$ is the total number of fingers ($N_f=5$ in this study). We used the statistics in (Lee et al. 2008) to calculate the joint angle when the hand is at rest pose.

#### Adaptation to human arm model

The manipulability measure $\omega ^{a}$ and task compatibility measure $\alpha _a$ of the arm model follow the measure defined in Eq. (2) and Eq. (4), respectively, with $\varvec{J}$ being the kinematic Jacobian of the arm. Comfort measure is modified for the arm model as:

9 $$\begin{aligned} \epsilon _a = 1-\frac{1}{N_{J}}\sum _{i=1}^{N_J} \bigg (\frac{q_i - q_i^r}{Q_i}\bigg )^{2},~i=1,2,\dots ,N_{J}, \end{aligned}$$

where $N_J$ is the number of arm DoFs ($N_J=7$ for each arm in this study), $q_i$ is the angle of $i\mathrm{th}$ joint, $q_i^r$ is the rest position angle of $i\mathrm{th}$ joint, and $Q_i$ is the corresponding joint limit.

### Understanding human kinematic coordination based on inverse optimization approach

We exploited inverse optimization to analyze the human upper limb model, in order (1) to infer the composition structure of the central nervous system’s optimal criteria, and (2) to understand how does this composition structure vary as human subject improves task proficiency.

#### Inverse optimization problem formulation

The inverse optimization problem (IOP) aims at identifying the formulation of the optimization problem (OP), i.e., inferring the form of objective function (or a combination of cost components), subject to which the OP can best reproduce the given optimal solutions (Tarantola 2005).

IOP assumes that the optimal objective function can be represented as a combination of multiple basic costs. The contribution of each basic cost to the entire composite criterion is represented by its corresponding weight coefficient. Weight coefficients can be identified by formulating the IOP as a bi-level optimization problem (Colson et al. 2007):

10 $$\begin{aligned} \begin{array}{rl} \text {Upper level problem} &{} \\ \min _{\varvec{\omega }} \varvec{\varPhi }(\varvec{x},~\varvec{x}^{*}), &{} \\ \text {s.t.~} \sum _{i=1}^{N}\omega _{i} = &{} 1,~\omega _{i}\in [0,1],~i=1,2,\dots ,N,\\ &{} \Updownarrow \\ \text {Lower level problem} &{} \\ \min _{\varvec{x}} \varvec{\varPsi }(\varvec{x}|\varvec{\omega }) = &{} \min _{\varvec{x}} \sum _{i=1}^{N}\omega _{i}~{\Psi }_{i}(\varvec{x}), \\ \text {s.t. } \varvec{g}_{j}(\varvec{x}) \le &{} 0,~j=1,2,\dots ,m,\\ \varvec{h}_{k}(\varvec{x}) = &{} 0,~k=1,2,\dots ,n. \end{array} \end{aligned}$$

#### Upper level problem

The upper level problem aims at finding the optimal weight vector $\varvec{\omega ^{*}}$^3^ that minimizes the distance (measured by $\varvec{\varPhi }$) between the current optimal state $\varvec{x}$ (solution of the lower level problem) and the optimal observed state $\varvec{x}^{*}$ (recorded human data). The optimization variable $\varvec{\omega }$ contains coefficients $\omega _{i}$, which are assigned to the metrics ${\Psi }_{i}$ as defined in the lower level problem. The metric $\varvec{\varPhi }$ can be defined either in joint space (error in joint angles) or in Cartesian space (error in end-effector pose). We used the sum of mean square errors of joint angles, since the constrained IK guarantees a unique mapping between model joint angles and Cartesian positions, and computing $\varvec{\varPhi }$ in joint space also reduces computational complexity. Constraints of the upper level problem include (1) boundary constraints of each weight coefficient $\omega _{i}\in [0,1]$, and (2) linear constraint $\sum _{i=1}^{N}\omega _{i} = 1$.

#### Lower level problem

The lower level problem is a direct optimization problem, and its target is to find the state $\varvec{x}$ that optimizes^4^ the objective function $\varvec{\Psi }$, which is assumed to be a linear combination of *N* basic costs ${\Psi }_i$, weighted by the corresponding coefficients $\omega _i$. We accounted the kinematic metrics described in Sect. 2.6, which we used to analyze the coordination patterns of hand and arm models, as high-level cost components ${\Psi }_i$, i.e., ${\Psi }_1=\omega ^{a}$, ${\Psi }_2=\alpha ^{a}$, and ${\Psi }_3=\epsilon ^{a}$.

It is noteworthy that the calculated kinematic measures cannot be combined directly, since they have different units and scales. To handle this problem, we introduced a normalization coefficient $\kappa _{i}$ to normalize the kinematic measures:

11 $$\begin{aligned}&\varvec{\varPsi }(\varvec{x}|\varvec{\omega }) = \sum _{i=1}^{N}\omega _{i}~\kappa _{i}{\Psi }_{i}(\varvec{x}), \end{aligned}$$

12 $$\begin{aligned}&\kappa _{i} = {\max \{{\Psi }_{i}(\varvec{x})\}}^{-1},~\varvec{x}\in [\underline{\varvec{x}}, \overline{\varvec{x}}],i=1,2,\dots ,N \end{aligned}$$

with $\underline{\varvec{x}}$ and $\overline{\varvec{x}}$ denoting the element-wise lower and upper boundaries of state variable $\varvec{x}$, respectively. The maximum value of each kinematic measure ${\Psi }_{i}$ is determined by optimizing ${\Psi }_{i}$ within the bounds of states.

To determine the state variable $\varvec{x}$ of the lower level optimization problem, we performed Sobol analysis to analyze the global sensitivity of cost ${\Psi }_{i}$ to each joint of the model. The analysis was conducted using the GSAT [Global Sensitivity Analysis Toolbox, Cannavó (2012)], and each joint was sampled within its motion range using quasi-random Monte Carlo method. Joint with a sensitivity ratio above $5\%$ was considered to have significant influence on the kinematic measures and then considered as one state variable.

The feasible movement range of each arm joint is limited both inherently by the biomechanical constraints and extrinsically by the task demands. For instance, in watchmaking, subjects usually place their forearms on the table and close to the watch face; consequently, the extension/flexion of the upper arm is limited. This is revealed by a low variance of the joint angle cross manipulation trials of subjects. Therefore, to better represent the changes in comfort feeling that are caused by the movements of arm joints, we weighted the comfort value of each model joint by a factor $\beta _{j}$:

13 $$\begin{aligned} \beta _{j} = \sqrt{\mathrm {Var}(\{\varvec{q}^{s}_{j}\})},~\varvec{q}^{s}_{j}\in [\underline{\varvec{q}}_{j},\overline{\varvec{q}}_{j}],~s=1,2,\dots ,N_{s}, \end{aligned}$$

where $N_{s}$ is the total number of subjects, $\varvec{q}^{s}_{j}$ is the $j\mathrm{th}$ joint angle of $s\mathrm{th}$ subject, $\underline{\varvec{q}}_{j}$ and $\overline{\varvec{q}}_{j}$ are the element-wise lower and upper boundaries of $\varvec{q}^{s}_{j}$, respectively. Thus $\beta _{j}$ is the standard deviation of $j\mathrm{th}$ joint angle cross all subjects. Values of $\beta _{j}$ are normalized cross joints in calculation, i.e., $\beta ^{\prime }_{j}=\beta _{j}/\sum _{j=1}^{N_{J}}\beta _j$ with $N_J$ being the total number of joints. Hence the comfort measure as defined in Eq. 9 is modified as:

14 $$\begin{aligned}&\epsilon ^{a} = 1-\frac{1}{N_J}\sum _{j=1}^{N_J}\beta ^{\prime }_{j}\bigg (\frac{q_j - q_j^r}{Q_j}\bigg )^{2},~j=1,2,\dots ,N_{J},\nonumber \\&\quad \sum _{j}^{N_{J}}\beta _{j}=1. \end{aligned}$$

Constraints of lower level problem include boundary constraints of each state variable. Joint angle $q_{i}$ is bounded within its corresponding anthropomorphic motion range, and rotation angle of the watch face $\theta$ is constrained within $[-\pi ,\pi ]$. Optimization problems of both levels were solved using derivative-free optimization method, since common gradient-based optimization approached may fail in this case due to the non-smooth nature of the objective function $\varvec{\varPhi }(\varvec{x},~\varvec{x}^{*})$. We used surrogate optimization approach (Gutmann 2001) in MATLAB Optimization Toolbox$^{\mathrm{TM}}$. Surrogate optimization is a global derivative-free approach for bounded optimization problems. It samples to approximate objective functions and is best suited to time-consuming computation. Global optimal solution is often difficult to guarantee in bi-level optimization problems, as the bi-level optimization framework is intrinsically restricted by its non-convex and nonlinear formulation (Bard 2013), and no efficient method has been proposed to solve this problem so far. We ran the inverse optimization problems five times for each subject and used the solution that results in the best objective function values. The weight $\varvec{\omega }_{i}$ was normalized before each lower level problem process starts, and hence the total cost of the lower level $\varvec{\Psi }(\varvec{x}|\varvec{\omega })$ was normalized within [0, 1]. The finally obtained optimal weight vector $\varvec{\omega }^{*}$ of each subject was normalized and used for analysis afterward.

**Fig. 4:**
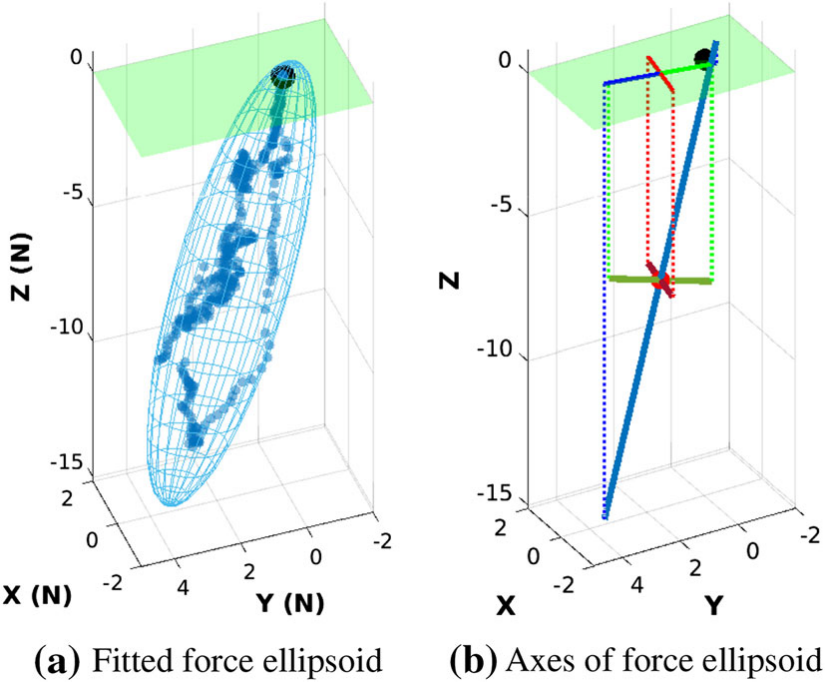
Example of a force ellipsoid and its axes extracted from an expert subject. The major axis of the force ellipsoid indicates the task-demanded direction of force application

**Fig. 5:**
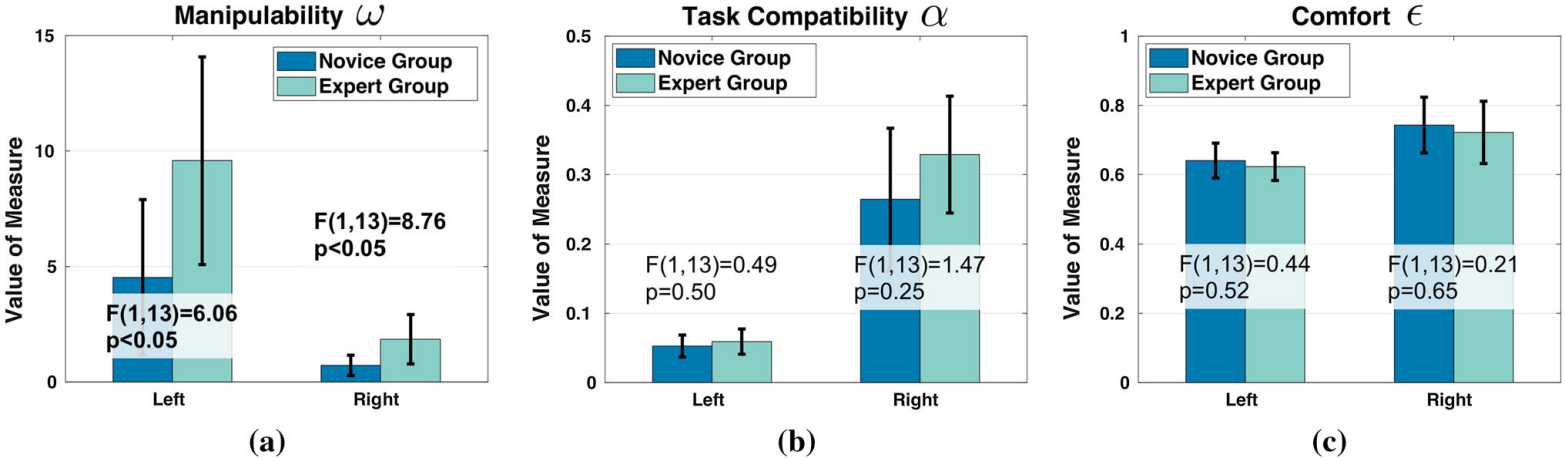
Kinematic measures of hands

## Results

### Extraction of task demands

We applied the Minimum Volume Enclosing Ellipsoid (MVEE) approach (Moshtagh et al. 2005) to fit the force ellipsoid. The optimal task-demanded force direction extracted from task force ellipsoids is $\varvec{f}^{*} = (-0.09\pm 0.09, 0.19\pm 0.09, -0.97\pm 0.02)^{\intercal }$, as Fig. 4 shows. Thus we used the unit vector $\varvec{u}^{*}_\mathrm {L}\triangleq \varvec{f}^{*}_\mathrm {XY}=(0,0,-1)^{\intercal }$ for left hand, and the normalized component on the horizontal plane, $\varvec{u}^{*}_\mathrm {R}\triangleq \varvec{f}^{*}_\mathrm {Z}=(-0.42,0.91,0)^{\intercal }$ for right hand, respectively.

### Analysis of hand poses

The kinematic metrics ($\omega ^{h}$, $\alpha ^{h}$, and $\epsilon ^{h}$) introduced in Sect. 2.7.1 are calculated for both hands of each subject in hand coordinate frame, which overlaps the end-effector coordination frame (at hand center position) of the arm model. One-way ANOVA was applied to compare performance of subjects in the two groups.

Task compatibility measure $\alpha ^{h}$ and comfort measure $\epsilon ^{h}$ are not significantly different between groups for both hands. The two groups differ only in the singularity metric $\omega ^{h}$ of hands. Experts have significant larger manipulability metric values than novice subjects for both hands (see Fig. 5a).

Inter-limb analysis revealed significance between hands within group. For singularity metric $\omega ^{h}$, both groups have much larger values in left hand ($\omega ^{h}_{N,L}=4.53\pm 3.36$, $\omega ^{h}_{E,L}=9.58\pm 4.50$) compared to right hand ($\omega ^{h}_{N,R}=0.72\pm 0.44$, $\omega ^{h}_{E,L}=1.85\pm 1.07$) at 0.01 significance level (see Table 1). While for task compatibility measure $\alpha ^{h}$, measures of right hands ($\alpha ^{h}_{N,R}=0.26\pm 0.10$, $\alpha ^{h}_{E,R}=0.33\pm 0.08$) are much larger than left hands ($\alpha ^{h}_{N,L}=0.05\pm 0.02$, $\alpha ^{h}_{E,L}=0.06\pm 0.02$) for both groups. Subjects in novice group show slightly larger comfort metric $\epsilon ^{h}$ in right hand ($\epsilon ^{h}_{N,R}=0.74\pm 0.08$) than in left hand ($\epsilon ^{h}_{N,L}=0.64\pm 0.05$). This difference is not significant in expert group ($\epsilon ^{h}_{E,L}=0.62\pm 0.04$, $\epsilon ^{h}_{E,R}=0.72\pm 0.09$).

**Table 1:** Inter-limb comparison of hand kinematic measures for both groups

	$\omega ^{h}$		$\alpha ^{h}$		$\epsilon ^{h}$	
	*p*	*F*	*p*	*F*	*p*	*F*
N	$<\mathbf{0 }.\mathbf{01 }$	$\mathbf{12 }.\mathbf{64 }$	$<\mathbf{0 }.\mathbf{01 }$	$\mathbf{41 }.\mathbf{46 }$	$<\mathbf{0 }.\mathbf{01 }$	$\mathbf{11 }.\mathbf{67 }$
E	$<\mathbf{0 }.\mathbf{01 }$	$\mathbf{13 }.\mathbf{99 }$	$<\mathbf{0 }.\mathbf{01 }$	$\mathbf{48 }.\mathbf{78 }$	0.055	5.03

N: novice group, degrees of freedom (1, 18). E: expert group, degrees of freedom (1, 8)

**Fig. 6:**
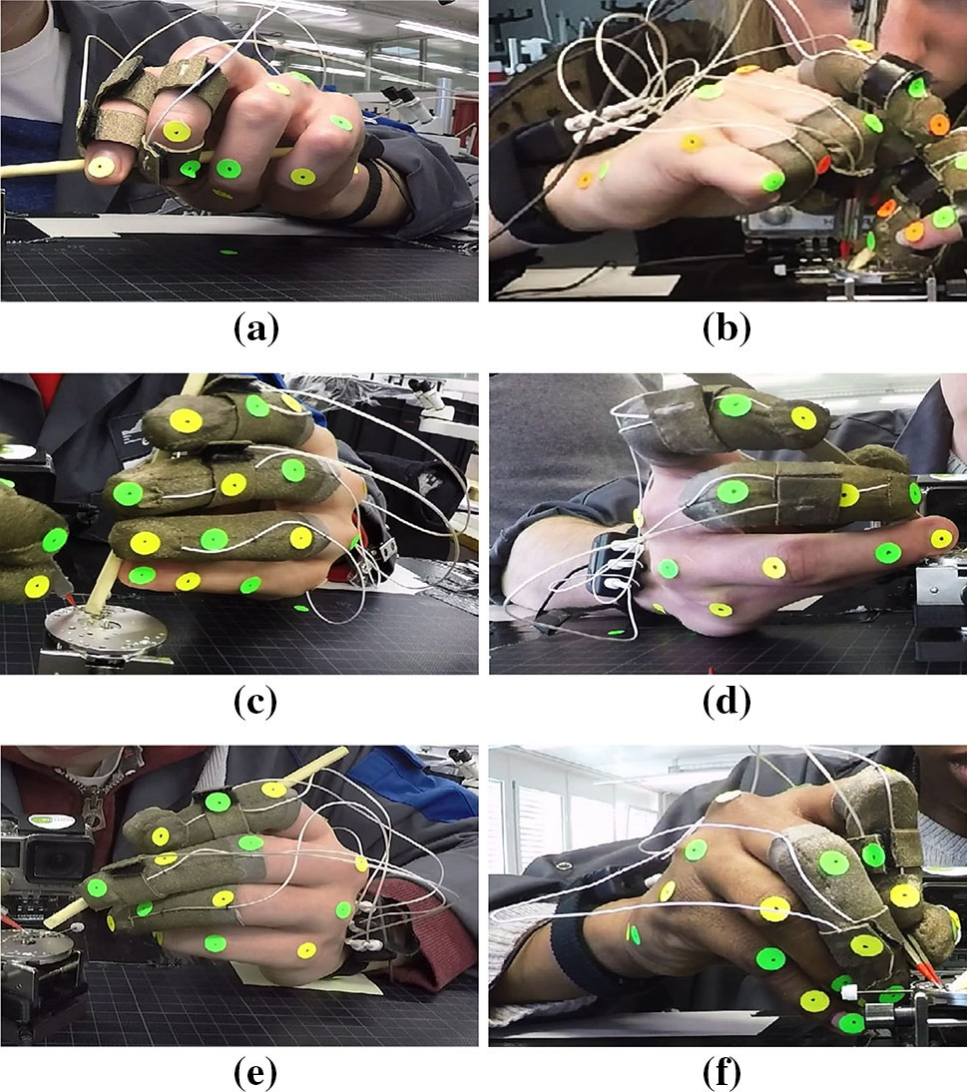
Comparison of typical hand poses

To gain more insights on this point, we compared hand poses employed by subjects during manipulation, as Fig. 6 shows.

**Fig. 7:**
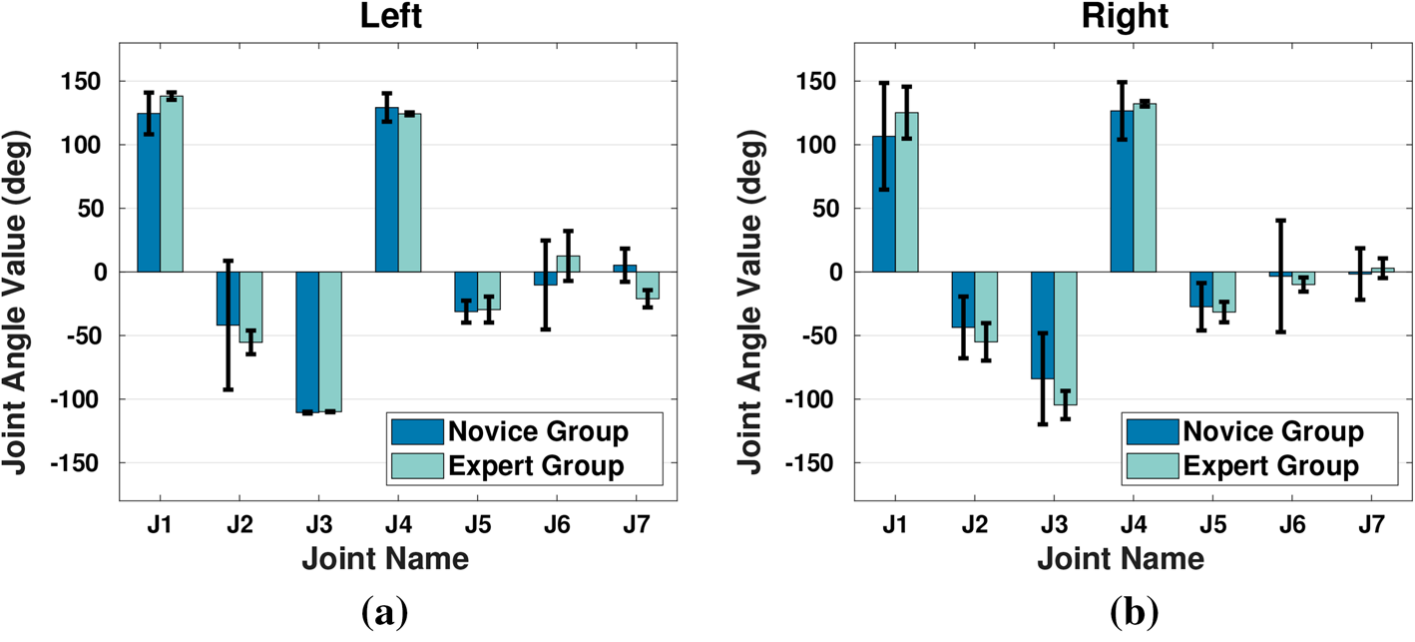
Joint angles of arm model obtained by solving the IK problem. Height of each bar indicates the mean joint angle value, and the error bar indicates the standard deviation

For left hand, most novice subjects prone to use hand poses close to either pose I-Left (Fig. 6a, fingers encompass the tool) or pose II-Left (Fig. 6c, thumb and other fingers press oppositely). In contrast, expert subjects are more likely to use hand pose III-Left (Fig. 6e to manipulate the pegwood. The differences across hand poses is revealed in the joint postures of each hand. Expert subjects have slightly larger angle ($35.87^{\circ }\pm 9.54^{\circ }$) in the extension/flexion DoF of index fingers’ DIP joint than novice subjects ($21.86^{\circ }\pm 8.87^{\circ }$) [$F(1,13)=7.94,~p<0.05$], while smaller angles of the extension/flexion DoF of the PIP joint for the ring finger [novice: $50.61^{\circ }\pm 19.47^{\circ }$, expert: $19.29^{\circ }\pm 17.55^{\circ }$, $F(1,13)=9.16,~p<0.01$] and the little finger [novice: $52.52^{\circ }\pm 19.53^{\circ }$, expert: $22.78^{\circ }\pm 27.18^{\circ }$, $F(1,13)=6.00,~p<0.05$]. For the right hand, six out of ten novice subjects have hand poses I-Right (Fig. 6b) or II-Right (Fig. 6d), and the others use hand pose III-Right (Fig. 6f). In comparison, most expert subjects use hand pose III-Right. No significant difference in right hand joints is observed.

**Fig. 8:**
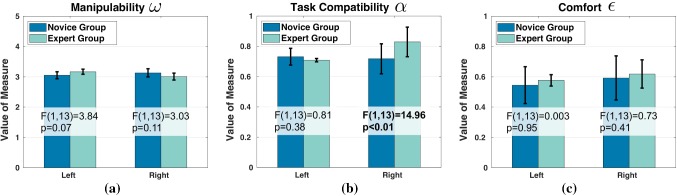
Kinematic measures of arms

### Analysis of arm postures

We compared the joint angle values of the arm model of all subjects. Subjects from both groups do not differ in arm postures (see Fig. 7) and have similar kinematic measure values in terms of manipulability (Fig. 8a) and comfort (Fig. 8c). However, the right arm configurations of expert subjects have a larger force transmission ratio ($\alpha ^{a}_{E,R}=0.83\pm 0.09$) than the novice subjects ($\alpha ^{a}_{N,R}=0.69\pm 0.04$) on average, at 0.01 significance level (see Fig. 8).

**Fig. 9:**
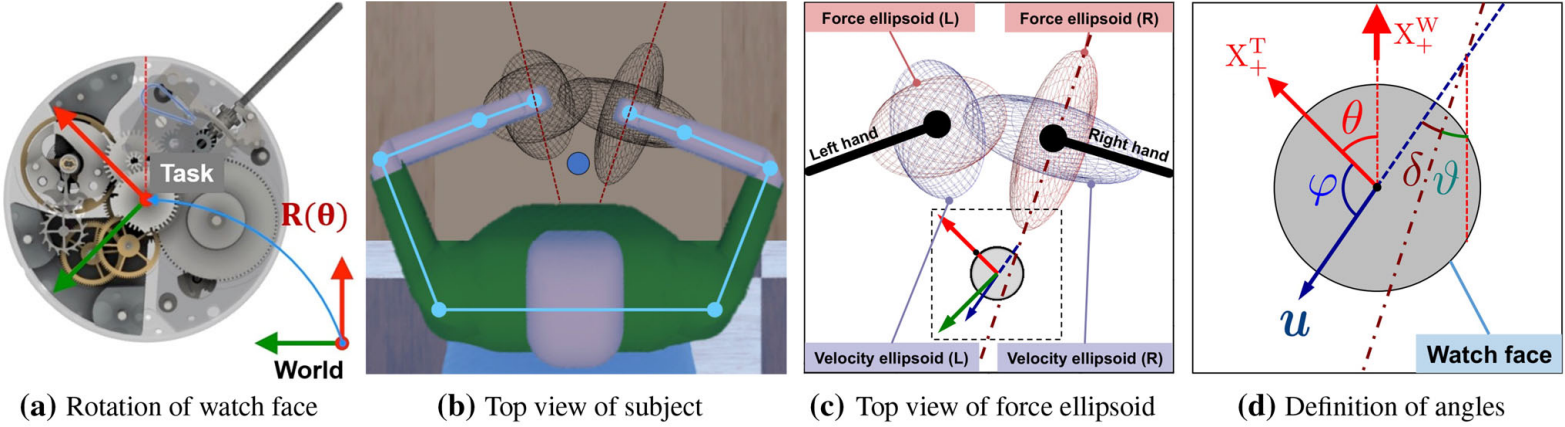
Geometrical factors used in the analysis. Force/velocity ellipsoids are projected onto the $\mathrm {X}_\mathrm {W}-\mathrm {Y}_\mathrm {W}$ plane for a better visualization of their major axes. $\theta$ is the angle to rotate ${\mathbb {W}}_\mathrm {CF}$ to ${\mathbb {T}}_\mathrm {CF}$. The task-demanded force application vector $\varvec{u}$ has an included angle $\varphi$ with $\mathrm {X}^{+}_\mathrm {T}$. $\vartheta$ is the included angle between the major axis of the project force ellipsoid and $\mathrm {X}^{+}_\mathrm {W}$. $\delta$ is the included angle between $\varvec{u}$ and the major axis of the projected force ellipsoid

This difference also holds for the projected 2D component of force ellipse’s major axis on the $\mathrm {X}-\mathrm {Y}$ plane (Fig. 9b, c). For left arm, novice group has an $\alpha _a$ value of $0.72\pm 0.03$ and expert group $0.71\pm 0.02$ [$p=0.37,~F(1,13)=0.98$]. For right arm, the $\alpha _a$ value is $0.69\pm 0.05$ for novice group, and $0.81\pm 0.10$ for expert group. This difference is observed at 0.05 significance level [$p=0.0127$, $F(1,13)=8.54$].

Inter-limb comparison indicates that expert subjects have slightly larger manipulability index in left arm ($\omega ^{a}_{E,L}=3.17\pm 0.08$, $\omega ^{a}_{E,R}=3.01\pm 0.11$), while larger task compatibility in right arm ($\alpha ^{a}_{E,L}=0.71\pm 0.01$, $\alpha ^{a}_{E,R}=0.83\pm 0.09$), both at 0.05 level (see Table 2). The comfort measure does not differ between limbs ($\omega ^{a}_{E,L}=0.58\pm 0.01$, $\omega ^{a}_{E,R}=0.66\pm 0.04$).

No inter-limb difference is observed in novice group, see Table 2 (manipulability: $\omega ^{a}_{N,L}=3.05\pm 0.12$, $\omega ^{a}_{N,R}=3.13\pm 0.13$; task compatibility: $\alpha ^{a}_{N,L}=0.73\pm 0.06$, $\alpha ^{a}_{N,R}=0.69\pm 0.04$; comfort: $\epsilon ^{a}_{N,L}=0.58\pm 0.07$, $\epsilon ^{a}_{N,R}=0.59\pm 0.15$).

**Table 2:** Inter-limb comparison of arm kinematic measures for both groups

	$\omega ^{a}$		$\alpha ^{a}$		$\epsilon ^{a}$	
	*p*	*F*	*p*	*F*	*p*	*F*
N	0.18	1.94	0.71	0.14	0.44	0.62
E	$<\mathbf{0 }.\mathbf{05 }$	$\mathbf{6 }.\mathbf{28 }$	$<\mathbf{0 }.\mathbf{05 }$	$\mathbf{7 }.\mathbf{52 }$	0.38	0.86

N: novice group, degrees of freedom (1, 18). E: expert group, degrees of freedom (1, 8)

### Alignment between limb postures and task demands

**Fig. 10:**
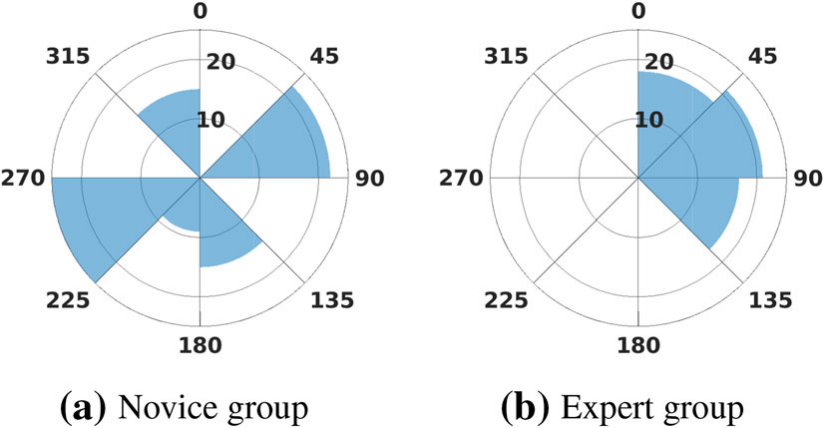
A summary of watch face rotation angles $\theta$. Radius indicates the number of motion trials that fall into this bin

To identify the factors that affect the task compatibility, we analyzed the rotation angle of watch face, which affects the task-demanded direction $\varvec{u}$. The angle $\theta$ is estimated from the pose of watch face (Fig. 9a). Slight divergence ($|\varDelta \theta |<10^{\circ }$) in $\theta$ is observed cross motion segments. To better illustrate the distribution of $\theta$, the mean values of $\theta$ in all motion segments from both groups were classified into eight main directions as Fig. 10 shows. We analysed subjects’ postures by calculating three angles: (1) the rotation angle of watch face $\theta$, (2) the optimal force application direction $\vartheta$ (the included angle between the major axis of force ellipsoid and $\mathrm {X}^{+}_\mathrm {W}$), and (3) the angle $\delta$ between the task-demanded force application direction $\varvec{f}^{*}$ and the major axis of the force ellipsoid. A geometrical illustration of these quantities is provided in Fig. 9d, and the joint angles are compared in Table 3. No significant difference is observed in $\vartheta$. This indicates that the optimal force application directions are similar in both groups. However, $\theta$ is different between two groups at 0.05 significance level. A significant difference in values for $\delta$ is also observed between groups [$F(1,13)=15.12,~p<0.01$].

**Table 3:** Kinematic coordination angles (in degree) of both arms under task demands (L: left, R: right)

	$\theta$	$\vartheta$ (L)	$\vartheta$ (R)	$\delta$ (L)	$\delta$ (R)
Novice Group	$-\,43.88\pm 74.93$	$-\,60.21\pm 48.99$	$-\,137.46\pm 40.41$	$88.96\pm 14.03$	$70.48\pm 20.41$
Expert Group	$39.91\pm 35.36$	$-\,44.84\pm 12.02$	$-\,151.24\pm 18.64$	$85.63\pm 17.65$	$29.74\pm 2.31$
*p*	$<\mathbf{0 }.\mathbf{05 }$	0.50	0.48	0.54	$<\mathbf{0 }.\mathbf{01 }$
*F*(1,13)	$~\mathbf{5 }.\mathbf{48 }$	0.46	0.51	0.40	$~\mathbf{15 }.\mathbf{12 }$

Notice that $\theta$ is directed angle ($\theta >0$ for anticlockwise rotation and $\theta <0$ for clockwise rotation). $\delta$ is the included angle between major axis of force ellipsoid and $\varvec{u}^{*}$ and is undirected. We calculate $\delta$ in $[0,90^{\circ }]$ due to the symmetry of major axes of force ellipsoid. The calculation of $\varphi$ uses the extracted task-demanded vector $\varvec{u}^{*}\triangleq \varvec{f}^{*}$ and is obtained as $\varphi =114.83^{\circ }$ (in the task coordinate frame)

**Fig. 11:**
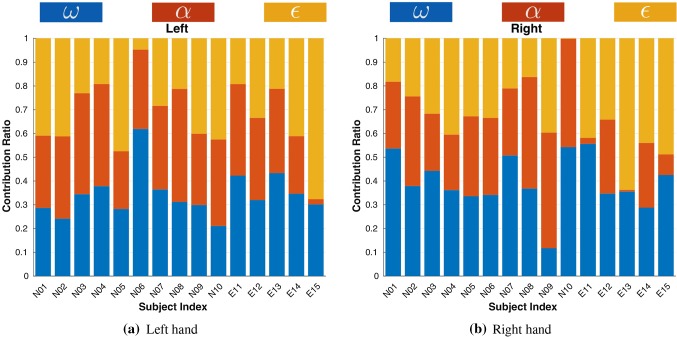
Normalized cost weights calculated for each subject. The prefix of subject number “N” denotes the novice subject (N01-N10), and “E” denotes expert subject (E11-E15)

### Identification of optimal criterion

Global sensitivity analysis suggests that sensitive model joints include arm circumduction (joint 2), forearm extension/flexion (joint 4), extension/flexion (joint 5), and supination/pronation (joint 6) for both arms. These DoFs are considered as state variables in the bi-level optimization problem. Besides, we consider the rotation angle $\theta$ as one state variable. We use the human joint angles found through inverse kinematics as the default values for unused joint angles. The lower level of the IOP converges within $150\sim 200$ iterations (stopping criterion: objective function value stopped decreasing, namely decrease is lower than $e^{-5}$ and the objective function has reach a low enough value, namely within an order of magnitude of $-1$). The upper level optimization problem is evaluated for 500 trials.

The weights obtained by solving the IOP for the objective function of each subject are reported in Fig. 11. Notice that the values are normalized and only indicate relative ratios instead of absolute weights of each cost. An intuitive explanation of a large weight is that the subject makes more effort to reduce/increase the corresponding cost, compared to other costs.

**Fig. 12:**
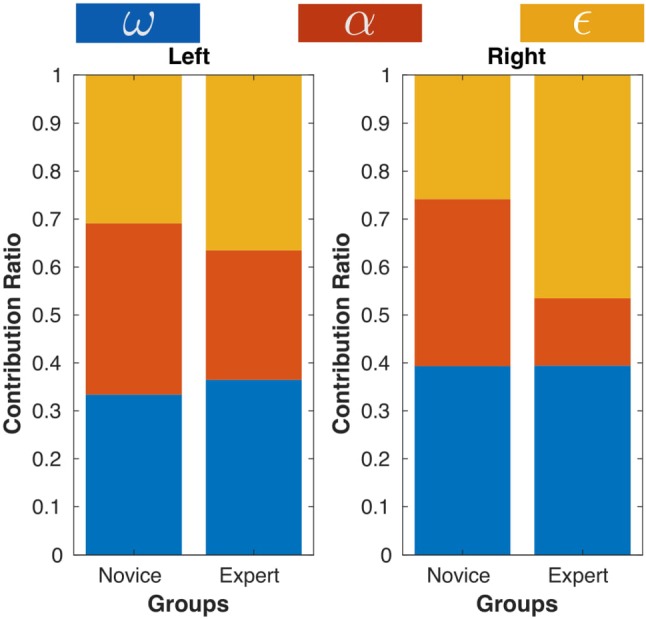
Average cost weights of both groups

Figure 12 illustrates the average weights for both groups. For the novice group, major contribution to overall cost measures comes from task compatibility ($c^{\alpha }_{N,L}=35.73\%\pm 6.98\%$) and manipulability ($c^{\omega }_{N,L}=33.38\%\pm 11.28\%$) for the left arm, while manipulability ($c^{\omega }_{N,R}=39.31\%\pm 12.58\%$) and task compatibility ($c^{\alpha }_{N,R}=34.86\%\pm 9.45\%$) for the right arm.

Cost related to increasing comfort is, however, less important for both arms ($c^{\epsilon }_{N,L}=30.89\%\pm 13.68\%$, $c^{\epsilon }_{N,R}=25.83\%\pm 12.38\%$). Expert subjects have similar contributions to overall cost for left arm ($c^{\omega }_{E,L}=39.31\%\pm 12.58\%$, $c^{\alpha }_{E,L}=34.86\%\pm 9.45\%$, and $c^{\epsilon }_{E,L}=25.83\%\pm 12.38\%$). However, for right arm, the major cost related to increasing comfort ($c^{\epsilon }_{E,R}=46.53\%\pm 10.99\%$), which is much higher than novice groups, indicated by a difference at 0.01 significance level [$F(1,13)=9.97,~p<0.01$, see Table 4]. The task compatibility cost has a much smaller contribution ($c^{\alpha }_{E,R}=14.06\%\pm 14.20\%$) than that of the novice group [$F(1,13)=11.65,~p<0.01$]. The contribution of manipulability [$c^{\omega }_{E,R}=39.41\%\pm 10.28\%$] is rather similar to novice group [$F(1,13)=0.0003,~p=0.99$].

**Table 4:** Comparison of calculated weight coefficients of kinematics costs between groups (L: left, R: right)

Measure	$\omega$		$\alpha$		$\epsilon$	
Arm	L	R	L	R	L	R
*p*	0.58	0.99	0.14	$<\mathbf{0 }.\mathbf{01 }$	0.52	$<\mathbf{0 }.\mathbf{01 }$
*F*(1,13)	0.32	0.0003	2.49	$~\mathbf{11 }.\mathbf{65 }$	0.43	$~\mathbf{9 }.\mathbf{97 }$

## Discussion and conclusions

The present study focused on the human kinematic coordination patterns in a bimanual fine manipulation task. The task requires coordination of both hands and arms in a master–slave relationship, resulting in motions of asymmetric and non-cyclic nature. Kinematics of upper limbs (hand poses, arm postures) play a crucial role in human bimanual motions, and postures seem to be planned in advance rather than fortuitous (Grea et al. 2000). As we saw in our study, experts spent less time on task execution than novices on average (expert: $45.88\pm 36.46$s, novice: $84.36\pm 54.86$s), but dedicated more time on adjusting hand/arm postures and poses of watch faces before manipulation (expert: $9.57\pm 6.76$s, novice: $6.38\pm 4.79$s). This shows that expert subjects prepare better and end up with efficient execution, indicating an understanding of the critical role of motion kinematics.

The results of our analysis demonstrate the influence of extrinsic constraints (i.e., task demands) in the generation of coordination patterns within the dynamic-pattern theoretical framework (Schöner and Kelso 1988; Kelso 1997). Experiments show that subjects learn to take advantage of task conditions as they improve their skills, which provide evidence to support the influential three-stage motor learning scheme proposed by Bernstein (1967), i.e., as expertise increases, the motor controller first freezes redundant DoFs, then releases the frozen DoFs, and finally uses external forces instead of struggling against them.

### Dexterity: a marker of skill

The “dexterity” of human hand is a rather abstract concept that encompasses multiple aspects with which one can associate multiple metrics (Kim and Khosla 1991). In this study, we used the manipulability index as a metric of dexterity, as it reveals the intrinsic ability of moving fingers to manipulate tools. Our analysis indicates a higher hand manipulability in expert subjects than in novice subjects on average (Fig. 5). We attributed differences in manipulability across groups to the distinct hand poses. Hand pose I (Fig. 6) is power grasp that guarantees a strong stability of the held tool. Groups differed also in the distribution of contact along fingers and tools: the tool is en-wrapped by fingers in pose I, while mainly contacted only by fingertips in pose II, which provide higher mobility of the tool. In contrast, hand pose III (mainly used by experts) requires primarily contact on thumb, index, and middle fingers, providing a higher flexibility of fingers (Fig. 5a) and is more suitable for dexterous manipulation. This hand pose is similar to the pose used for grasping a pencil to write, as discussed in Kelso and Clark (1982) and Li and Sastry (1988). It hence seems that as subjects accumulate more knowledge of the task as a result of practice, they tend to modulate their finger placements to increase the dexterity. They move away from highly stable power grasp poses to poses that ensure a higher flexibility of the tool.

Comparison of left and right hand poses within group reveals significant differences of kinematic properties, as a result of distinct hand poses. Subjects in both groups used more fingertip grasping poses for holding pegwood (left hand). In contrast, more contacts on intermediate and proximal phalanges were made to enfold the tweezers (right hand). Fingertip contacts enable higher flexibility of finger motion, correspond to a higher manipulability measure $\omega _h$ of left hands on average. This selection of hand pose is essentially determined by the requirements of manipulating the tools. Left hand poses of higher $\omega _h$ enable flexible adjustment of pegwood to maintain the watch spring stable, while right hand poses of lower $\omega _h$ guarantee the stability of manipulating tools by restricting the finger motion.

The significant inter-limb differences of hand poses in both groups indicate that the selection of hand poses depends largely on the manipulation tools, thus highly task-oriented. This finding provides evidence for the task-based grasp planning of human CNS and in consistency with the study of Friedman and Flash (2007). Furthermore, the difference observed between groups implies the influence of skill levels (or task proficiency) on hand pose selection. Expert subjects manifest the flexibility of placing fingers on the tools (indicated by significant higher $\omega _h$ value than novice group), under the premise of maintaining the ability to satisfy task demands (slightly larger $\alpha _h$ than novice group). We thus conclude that the coordination mechanisms exploited by human to control fingers for tool use is not only affected by the potential functionality provided by the hand pose, but also influenced by skill level, which reflects subject’s understanding of the task demands.

It is noteworthy that although power grasps better stabilize the tools, they do not guarantee higher task compatibility (see Fig. 5b). The reason is that the metric $\alpha$ only reveals the ability to transfer joint torque to operational space force. If we consider the left hand pose as an example, increasing finger joint torque in power grasp (hand pose I) empowers the hand to grasp the tools more tightly, but does not generate larger force in the task-relevant direction. To compensate for the insufficient force generated by the hand, we observed that novices subjects would instead adjust arm postures (e.g., rotating wrist, the $6\mathrm{th}$ and $7\mathrm{th}$ joints, see Fig. 7) to increase the force generated by the hand. This is confirmed by the slightly higher $\alpha _a$ value and lower $\epsilon _a$ in novice group compared to the expert group (see Fig. 8b, c). Moreover, the adjustment of wrist joints also causes a lower manipulability of dominant arm (right) than assistant arm (left) as reported in expert group (Fig. 8a). It benefits the task execution in the sense that the dominant arm is less flexible in motion and obtain higher stability of holding tools as consequence. This is also consistent with the analysis of hand poses (Fig. 5a). The fact that experts’ hand pose allows for a better transmission of force in the task-relevant direction results in less demand on the arm. This reduction on arm joint efforts is beneficial particularly in manipulation tasks that can last hours, such as watchmaking. Our analysis indicates, hence, that difference in hand and arm pose across subjects is mainly to be attributed to task demands (direction of $\varvec{u}$) and, to a lesser extent, to the comfort of arm joint angles. Such goal-directed arm and hand movements are a particular characteristic of our results.

### Goal-directed coordination patterns

A few studies discussed generation of goal-directed coordination pattern. For instance, when reaching for grasping a moving target, arm and hand motions are tightly coupled to the target’s location and orientation (Jeannerod et al. 1995). Bingham et al. (2008) suggested that the coordinate timing of bimanual movements to distinct targets is biased toward synchrony and also influenced by visual information. In these studies, however, task demands (i.e., spatial position of the reaching target) are unaffected by human motions and “extrinsic” to the generated coordination patterns. In this sense, the generation of coordination is essentially an adaptation of the biomechanics of human subjects’ musculoskeletal system to the external task demands. In our study, subjects could actively change task demands by re-orienting the watch. This is revealed through an analysis of the distribution of $\theta$ (see Table 3). Results indicate that expert subjects take advantage of the fact that the watch can be re-oriented (see Fig. 3) and hence that the task demand can be modified to find a better alignment between task demand and task compatibility. Inconsistency with this finding is the higher manipulability ($\omega _a$) of left arm compared to right arm observed in expert group (see Table 2), indicating arm postures that enable higher motion flexibility.

We conclude that expert subjects differ from novice subjects in the sense that they exploit adjustable task conditions to “wisely” modify the task demands, i.e., they rotate the watch face toward a configuration that is more convenient for them to perform task, instead of adjusting their arm postures to adapt to the task demands. From this perspective, “coordination” in our study exceeds the notion of coordination across hand joints and arm joints and also entails coordination with the manipulated objects. This leads to the question of which reference frame is used for control and hence underpin coordination. As pointed out in Swinnen (2002), switching across reference frames helps to mediate multiple constraints of coordination. Task constraints may affect CNS’s choice of reference frames for movements encoding, such as egocentric (intrinsic) reference, or allocentric (extrinsic) reference (Swinnen et al. 1997). Our analysis therefore suggests that control of the arms may be performed in a frame of reference attached to the watch and that, prior to perform the task, the CNS computes an optimal re-orientation of the task frame of reference in global coordinates.

### Understanding acquisition of coordination under optimal control framework

The present study is unique in the sense that for the first time an inverse optimal control approach is used to understand the confluence of multiple constraints in the generation of kinematic coordination patterns in bimanual coordination. Unlike previous studies, in which (inverse) optimal control approach is exploited to analyze the movement principles of human motion, we consider the proficiency of human subjects and utilize IOP to infer changes in the constraints that affect the generation of coordination patterns. We make the assumptions that (1) the CNS searches for optimal solutions that balance both intrinsic and extrinsic constraints; and (2) the composition structure of this optimal balance reveals subject’s proficiency (i.e., skill level). It is likely that the weights associated with satisfying each constraint change as humans learn about the task and their ability to perform the task, such as increasing motion adaptability in addition to satisfying task constraints (Seifert et al. 2013). Nelson (1983) also pointed out that skilled movements satisfy more common constraints such as “economy of effort” or “efficiency” in addition to task-oriented objectives.

Comparing the optimal criterion’s composition structure between novice and expert groups, we observe that task compatibility cost only contributes a relative small part to the total cost on average in expert groups. However, this does not mean that experts ignore task demands. In fact, the average task compatibility index of expert subjects is higher than the novice subjects on average (Fig. 8). Task demand is hence more important in absolute terms but less than other costs for experts. We attribute this to experts’ ability to adjust to task demands, i.e., through a rotation of watch face (Table 3), as we discussed in previous section.

When comparing costs of right and left arm, we find no significant difference in the costs for the left (assistant) arm between groups. This is likely due to the fact that the left arm has invariant task demands despite the rotation of the watch face, since pressing the pegwood downwards does not require directional control on the horizontal plane. In contrast, the motion of the right arm is highly dependent to the task-relevant direction and thus we see higher weight for comfort cost for this arm in the expert group. This is consistent with the calculated kinematic metrics of arms (see Fig. 8c). Expert subjects have slightly higher comfort index values of both arms than novice subjects on average, although not significant. These results speak once more in favor of the hypothesis that novice subjects focus on achieving limb postures that can better satisfy task demands, while expert subjects aim at higher comfort feelings by avoiding stretching arm joints.

On the basis of our analysis, we hypothesize that during the acquisition process of novel skills, human subjects first focus on searching for arm postures that satisfy task demands as displayed by novice subjects. Afterward, as humans acquire task experience and improve their skills, they tend to use more comfortable gestures for task execution, which could be realized by taking advantage of task specifications and adjusting task demands (e.g., rotating watch face in this study). This result supports the learning scheme proposed by Bernstein (1967) as introduced at the beginning of our discussion, in particular the final stage–experts exploit external forces instead of fighting against them, since the performance of expert subjects clearly manifests that they take advantage of the external environmental conditions rather than to fight against them (Latash 2010). This is in accordance with the findings in Seifert et al. (2011), which shows that experts manifest more adjustment of environmental constraints than novices by exploring properties of frozen water falls during ice climbing. As a result, this leads to increases of both task compatibility and comfort feeling of the musculoskeletal system.

### Limitations

Notwithstanding, our current work has limitations that need better clarification.

First, we analyzed kinematic coordination patterns of hands and arms separately. On the one hand, the undirected metrics ($\omega$ and $\epsilon$), which are independent of task demands ($\varvec{u}$), can be calculated for hand and arm individually. It could be hard to imply that the singular postures and the comfort feeling of arm may affect the corresponding metrics of hands, and vice versa. On the other hand, no significant difference is founded in the metric $\alpha$ between groups, indicating that the ability to generate task-relevant force (task compatibility) is not affected by the hand poses. Therefore, also to formulate a computationally feasible problem, we did not consider finger joints in the formulation of the inverse optimization problem. Second, we did not consider individual differences during learning. It is, however, known that prior knowledge, personal experience and individual-specific abilities affect task performance (Ferguson 1956). Similarly, multiple factors, such as expertise, motivation and personality, may also affect the learning process (Williams and Hodges 2004). In the present study, we, however, ignored this aspect and hypothesized that the costs for novices and experts were the same and that learning would only result in a modulation of the weights.

Furthermore, we formulated our cost components at task level. Thus, it is arguable that the formulation of kinematic metrics may not reveal low-level costs for the human’s musculoskeletal system (Nelson 1983). The purpose of our study, however, is to interpret human arm–hand coordination generation at task level from multiple aspects (i.e., singularity aspect, task completion aspect, or biomechanical aspect). In this way, we also aim to reduce the correlation across low-level cost components. For example, costs that comprise joint angle accelerations $\ddot{\theta }$ and costs that comprise angle jerk $\dddot{\theta }$ (Berret et al. 2011). However, we cannot exclude other aspects, which are not taken into consideration in the present work, influence the task performance. Further decomposition of current task level costs may help to gain deeper insights into the actual structure of the CNS’s optimal criteria in motion planning and generation.

## Footnotes

1 We use superscripts (“$+$” and “−”) to denote directions of axes and subscripts (“$\mathrm {W}$” and “$\mathrm {T}$”) to indicate the coordinate frame.

2 We use superscription “h” and “a” to indicate the measures of hand and arm, respectively. The subscriptions “E” and “N” indicate the group, and “L” and “R” indicate the side. For example, $\omega ^{h}_\mathrm {E,L}$ represents the $\omega$ calculated value of left hand in expert group.

3 We use the superscription “$*$” to indicate the optimal value (solution) of the variable.

4 The objective function $\varvec{\Psi }$ is supposed to be maximized in the lower level problem. However, we still use “minimize” to maintain the consistency of description.
